# CRMP4 CpG Hypermethylation Predicts Upgrading to Gleason Score ≥ 8 in Prostate Cancer

**DOI:** 10.3389/fonc.2022.840950

**Published:** 2022-03-10

**Authors:** Xiao-Ping Qin, Qi-Ji Lu, Cheng-Huizi Yang, Jue Wang, Jian-Fan Chen, Kan Liu, Xin Chen, Jing Zhou, Yu-Hang Pan, Yong-Hong Li, Shan-Cheng Ren, Jiu-Min Liu, Wei-Peng Liu, Hui-Jun Qian, Xian-Lin Yi, Cai-Yong Lai, Li-Jun Qu, Xin Gao, Yu-Sheng Xu, Zheng Chen, Yu-Min Zhuo

**Affiliations:** ^1^ Department of Urology, The First Affiliated Hospital of Jinan University, Guangzhou, China; ^2^ Department of Urology, Affiliated Xiaolan Hospital, Southern Medical University, Zhongshan, China; ^3^ Department of Thoracic Surgery, The First Affiliated Hospital of Jinan University, Guangzhou, China; ^4^ Department of Pathology, The First Affiliated Hospital, Sun Yat-sen University, Guangzhou, China; ^5^ Department of Urology, The Third Medical Centre, Chinese People's Liberation Army (PLA) General Hospital, Beijing, China; ^6^ Department of Pathology, The Third Medical Centre, Chinese People's Liberation Army (PLA) General Hospital, Beijing, China; ^7^ Department of Pathology, The Third Affiliated Hospital, Sun Yat-sen University, Guangzhou, China; ^8^ Department of Urology, Cancer Center, Sun Yat-sen University, Guangzhou, China; ^9^ Department of Urology, The First Affiliated Hospital of Naval Medical University, Shanghai, China; ^10^ Department of Urology, Guangdong General Hospital, Guangzhou, China; ^11^ Department of Urology, The First Affiliated Hospital, Nanchang University, Nanchang, China; ^12^ Department of Urology, Renmin Hospital of Wuhan University, Wuhan, China; ^13^ Department of Urology, Cancer Hospital, Guangxi Medical University, Nanning, China; ^14^ Department of Urology, The Third Affiliated Hospital of Sun Yet-Sen University, Guangzhou, China; ^15^ Department of Emergency, The First Affiliated Hospital of Jinan University, Guangzhou, China

**Keywords:** prostate cancer, gleason score upgrade, CRMP4 promoter methylation, biochemical recurrence, pelvic lymph node dissection

## Abstract

**Background:**

This study determined the predictive value of CRMP4 promoter methylation in prostate tissues collected by core needle biopsies for a postoperative upgrade of Gleason Score (GS) to ≥8 in patients with low-risk PCa.

**Method:**

A retrospective analysis of the clinical data was conducted from 631 patients diagnosed with low-risk PCa by core needle biopsy at multiple centers and then underwent Radical Prostatectomy (RP) from 2014-2019. Specimens were collected by core needle biopsy to detect CRMP4 promoter methylation. The pathologic factors correlated with the postoperative GS upgrade to ≥8 were analyzed by logistic regression. The cut-off value for CRMP4 promoter methylation in the prostate tissues collected by core needle biopsy was estimated from the ROC curve in patients with a postoperative GS upgrade to ≥8.

**Result:**

Multivariate logistic regression showed that prostate volume, number of positive cores, and CRMP4 promoter methylation were predictive factors for a GS upgrade to ≥8 (OR: 0.94, 95% CI: 0.91-0.98, *P*=0.003; OR: 3.16, 95% CI: 1.81-5.53, *P*<0.001; and OR: 1.43, 95% CI: 1.32-1.55, *P*<0.001, respectively). The positive predictive rate was 85.2%, the negative predictive rate was 99.3%, and the overall predictive rate was 97.9%. When the CRMP4 promoter methylation rate was >18.00%, the low-risk PCa patients were more likely to escalate to high-risk patients. The predictive sensitivity and specificity were 86.9% and 98.8%, respectively. The area under the ROC curve (AUC) was 0.929 (95% CI: 0.883-0.976; *P*<0.001). The biochemical recurrence (BCR)-free survival, progression-free survival (PFS), and cancer-specific survival (CSS) were worse in patients with CRMP4 methylation >18.0% and postoperative GS upgrade to ≥8 than in patients without an upgrade (*P ≤* 0.002).

**Conclusion:**

A CRMP4 promoter methylation rate >18.00% in prostate cancer tissues indicated that patients were more likely to escalate from low-to-high risk after undergoing an RP. We recommend determining CRMP4 promoter methylation before RP for low-risk PCa patients.

## 1 Introduction

Prostate cancer (PCa) is one of the most common cancers affecting males, especially in developed countries (1). An accurate diagnosis of PCa can be made based on the prostate-specific antigen (PSA) level, digital rectal examination, radiographic examination, and core needle biopsy of the prostate gland. The Gleason score (GS) provides a reference for developing the treatment regimen and evaluating the prognosis. According to the National Comprehensive Cancer Network (NCCN) guidelines, patients diagnosed with low-risk PCa with the GS ≤6, T1-T2a and PSA<10ng/ml require active surveillance (AS) or radical prostatectomy (RP). Patients diagnosed with high-risk PCa with the GS≥8, ≥T3a or PSA>20ng/ml should undergo RP with pelvic lymph node dissection (PLND) (2). It has been reported (3–9) that 30%-55% of PCa patients developed a GS upgrade based on the postoperative pathologic evaluation; thus, they have already missed the best treatment regimen available. This is particularly the case for those with an escalation from low-to-high risk based on the postoperative pathologic evaluation. Such patients should have undergone RP plus PLND, while they only underwent AS or RP and therefore missed the best treatment regimen. Indeed, the question is whether low-risk PCa patients require core needle biopsies to predict the likelihood of a GS upgrade and optimize the treatment regimen before performing an RP.

Many factors have been proposed for the prediction of a GS upgrade: PSA level; prostate-specific antigen density (PSAD); body mass index (BMI); prostate volume; clinical T stage; the number of biopsies; the number of positive cores; percentage of positive cores; serum testosterone level; neutrophil-to-lymphocyte ratio; and type of biopsy technique (5, 10–14). There have been studies involving the use of biopsies to predict the escalation from low-to-high-risk PCa following an RP. It has only been reported (15) that the PSA level is correlated with a GS upgrade to ≥8. Studies have shown that DNA methylation is closely related to tumor progression (16, 17). The collapsin response mediator protein 4 (CRMP4) is a member of the CRMP family and is a tumor suppressor gene for prostate cancer metastases. Existing studies have demonstrated that CRMP4 promoter methylation leads to downregulation of CRMP4, thus promoting PCa invasion and metastases (18). Improving the diagnostic and treatment accuracy of PCa has become an urgent issue in the age of precision medicine. Herein we discuss the predictive value of CRMP4 promoter methylation in escalation decisions from low-to-high-risk PCa based on core needle biopsies. Other potential risk factors were also evaluated to optimize the treatment regimen before performing an RP.

## 2 Materials AND METHOD

### 2.1 Sources of Patients

A retrospective analysis of the clinical data was conducted from 631 patients diagnosed with low-risk PCa by core needle biopsies at multiple centers, then undergoing RP from 2014-2019. 61 and 570 patients with and without a postoperative GS upgrade to ≥8, respectively. The following data were collected from 631 PCa patients: age; PSA level; prostate volume; PSAD; the number of biopsies; the number of positive cores; percentage of positive cores; clinical T stage; pathologic T stage; GS based on core needle biopsy; GS upon postoperative pathologic evaluation; cut-off value for CRMP4 promoter methylation rate based on core needle biopsy; positive resection margins; seminal vesicle invasion; lymph node metastases; biochemical recurrence (BCR) and the time of BCR; clinical progression and the time of progression; and cancer-specific (CS) death and the time of CS death. Definition of GS grade was as follows: GS ≤ 6 (grade group1); GS=3+4(grade group2); GS=4+3 (grade group3); and GS≥8 (grade group4 or 5). An upgrade was considered if the grade group was higher in postoperative pathologic evaluation than preoperative core needle biopsy results (2, 19).

### 2.2 Follow-Up

The patients were followed once every 3 months in the 1^st^ year after surgery, then every 6 months in the 2^nd^ year. The follow-up was then performed annually. The follow-up evaluations included the following: BCR; clinical progression; and CS death. The definition of BCR was a PSA level ≥ 0.2 ng/ml on 2 consecutive determinations after the RP (20). The definition of clinical progression was a local recurrence or systemic metastases diagnosed by biopsy or radiographic evaluation (21). The definition of a CS death was a death caused by or related to PCa (22).

### 2.3 CRMP4 Promoter Methylation

Core needle biopsies collected prostate tissues from 631 patients, and paraffin-embedded samples were performed to detect CRMP4 CpG methylation. The paraffin-embedded samples were first used for pathologic evaluation before detecting CRMP4 CpG methylation. Based on the postoperative pathologic evaluation, the pathologist who established the diagnosis selected the cores with the highest GS. A laser microdissection system (Leica 6500; Germany) was used to label and dissect the cancer area (23). DNA was extracted from the tissues, amplified by PCR, and modified by hydrosulphite. Pyrosequencing was performed, and the primers used in the present study are described in our previous report (24, 25). Graphs showing the distribution of CRMP4 methylation are included, see the **Supplementary Materials** for details.

### 2.4 Inclusion Criteria

1. All patients had a GS ≤ 6, PSA level<10 ng/ml, and clinical T stage ≤T2a based on the preoperative biopsy; the number of biopsies was ≥8;
2. All patients underwent an RP, and a postoperative pathologic diagnosis was established;
3. All patients had complete clinical data, including preoperative indicators, postoperative pathologic findings, and follow-up evaluation findings;
4. All patients had core needle biopsies to collect tissue samples for CRMP4 promoter methylation detection.

### 2.5 Exclusion Criteria

(1). GS=7 based on postoperative pathologic evaluation;
(2). Deaths due to reasons other than PCa.

### 2.6 Statistical Analysis

Statistical analyses were performed using SPSS 27.0. Continuous data are expressed by ranges, frequencies are expressed by percentages, continuous variables were analyzed using t-tests; and categorical variables were analyzed using chi-square tests. Logistic regression was performed to identify the predictive factors for a postoperative GS upgrade to ≥8. Optimal cut-off values were determined from the ROC curves for potential predictive factors, including CRMP4 promoter methylation, and the more accurate predictive factor was identified. The BCR-free survival, progress-free survival, and CSS were calculated by Kaplan-Meier survival analysis for patients with high CRMP4 methylation and patients with low CRMP4 methylation. A *P*<0.05 was considered to indicate a statistically significant difference.

## 3 Result

### 3.1 General Features of Patients With a Postoperative GS Upgrade to ≥8

The average age of the 61 patients with a postoperative GS upgrade to ≥8 was 67 ± 7.1 years, a PSA level of 7.52 ± 2.26 ng/ml, and a PSAD of 0.25 ± 0.12 ng/ml^2^. For these patients, the prostate volume was 32.27 ± 11.78 ml, the total number of biopsies was 14 ± 4.8, the number of positive cores was 3 ± 1.2, the percentage of positive cores was 0.22 ± 0.09, and the median follow-up time was 57 ± 35.0 months (**Table 1**).

**Table 1:** General features of patients with a postoperative upgrade in GS to ≥8 and those without such an upgrade.

Variable	Total, NO. (%)		p-value
	GS ≤ 6 (GG=1)	GS≥8 (GG≥4)	
**No.of cases**	570	61	–
**Median age (y,range)**	66 (43-85)	67 (50-81)	0.468^a^
**Median PSA (ng/ml,range)**	7.89 (1.20-9.99)	7.15 (2.30-9.99)	0.017^a^
**Median prostate volume (ml,range)**	42.50 (10.01-243.02)	32.27 (12.36-65.16)	<0.001^a^
**Median PSAD (ng/ml**2**,range)**	0.23 (0.02-0.91)	0.25 (0.07-0.75)	0.377^a^
**No.of cores,median (range)**	14 (8-24)	14 (8-24)	0.959^a^
**Positive cores,median (range)**	2 (1-6)	3 (1-6)	<0.001^a^
**Percent positive core**	0.15 (0.05-0.38)	0.22 (0.04-0.38)	<0.001^a^
**CRMP4 value**	5.54 (0.00-25.71)	24.39 (3.86-47.00)	<0.001^a^
**Biopsy Gleason sum n**			–
≤6	570 (100%)	61 (100%)	
**Clinical T stage n**			<0.001^b^
T1	194 (34.04%)	7 (11.48%)	
T2a	376 (65.96%)	54 (88.52%)	
**Pathological T stage n**			<0.001^b^
T1	27 (4.74%)	0 (0%)	
T2a	279 (48.95%)	5 (8.20%)	
T2b	120 (21.05%)	7 (11.48%)	
T2c	92 (16.14%)	31 (50.82%)	
≥T3	52 (9.12%)	18 (29.51%)	
**Seminal Vesicle Invasion n**			<0.001^b^
Negative	561 (98.42%)	44 (72.13%)	
Positive	9 (1.58%)	17 (27.87%)	
**Surgical Margin n**			<0.001^b^
Negative	508 (89.12%)	36 (59.02%)	
Positive	62 (10.88%)	25 (40.98%)	
**Lymph node invasion n**			<0.001^b^
Negative	539 (94.56%)	47 (77.05%)	
Positive	31 (5.44%)	14 (22.95%)	
**Biochemical recurrence n**	88 (15.44%)	35 (57.38%)	<0.001^b^
**Clinical progression n**	38 (6.67%)	22 (36.07%)	<0.001^b^
**Death n**	13 (2.28%)	10 (16.39%)	<0.001^b^
**Follow-up Months,median (range)**	63 (7-124)	57 (8-124)	0.256^a^

Values are presented as mean (range) deviation or number (%) unless otherwise indicated.

GS, gleason score; GG, grade group; PSA, prostate specific antigen; PSAD, prostate specific antigen density; CRMP4, collapsin response mediator protein 4.

a Based on Student t-test.

b Based on chi-square test.

### 3.2 Comparison of General Preoperative Features Between Patients With and Without a Postoperative GS Upgrade to ≥8

Compared to patients without a postoperative GS upgrade, patients with a postoperative GS upgrade to ≥8 had a lower PSA level and a smaller prostate volume, but an increase in the number of positive cores, percentage of positive cores, CRMP4 promoter methylation rate, and more advanced clinical T stage. There were 61 and 570 patients with and without a postoperative GS upgrade to ≥8, respectively. There were no significant differences in age, PSAD, number of biopsies, and duration of follow-up between the two groups (*P*=0.468, *P*=0.377, *P*=0.959, and *P*=0.256, respectively). The mean PSA level was 7.89 ± 1.85 ng/ml in the patients without a GS upgrade compared to 7.15 ± 2.26 ng/ml in patients with a postoperative GS upgrade to ≥8 (*P*=0.017). The mean prostate volume was 42.50 ± 21.25 ml in patients without a GS upgrade compared to 32.27 ± 11.78 ml in patients with a postoperative GS upgrade ≥8 (*P*<0.001). The mean number of positive cores was 2 ± 0.9 in patients without a GS upgrade compared to 3 ± 1.2 in patients with a postoperative GS upgrade to ≥8 (*P*<0.001). The mean percentage of positive cores was 0.15 ± 0.06 in patients without a GS upgrade to 0.22 ± 0.09 compared to patients with a postoperative GS upgrade to ≥8 (*P*<0.001). The mean CRMP4 promoter methylation rate was 5.54 ± 2.75% in patients without a GS upgrade compared to 24.39 ± 10.34% in patients with a postoperative GS upgrade to ≥8 (*P*<0.001). The patients staged with cT2a disease accounted for 88.52% and 65.96% of all patients with and without a GS upgrade, respectively (*P*<0.001; **Table 1**).

### 3.3 Risk factors Predicting a Postoperative GS Upgrade to ≥8

#### 3.3.1 Analysis of Risk Factors Predicting a Postoperative GS Upgrade to ≥8 Based on Univariate and Multivariate Logistic Regression

Univariate logistic regression showed that the PSA level, prostate volume, number of positive cores, CRMP4 promoter methylation ra[te, and clinical T stage were factors predicting a postoperative GS upgrade to ≥8 (OR: 0.84, 95% CI: 0.74-0.95, *P*=0.005; OR: 0.96, 95% CI: 0.95-0.98, *P*<0.001; OR: 1.95, 95% CI: 1.52-2.49, *P*<0.001; OR: 1.40, 95% CI: 1.32-1.49, *P*<0.001; and OR: 3.98, 95% CI: 1.78-8.91, *P*=0.001, respectively). Multivariate logistic regression showed that prostate volume, number of positive cores, and the CRMP4 promoter methylation rate were all factors predicting a GS upgrade to ≥8. The positive predictive rate was 85.2%, the negative predictive rate was 99.3%, and the overall predictive rate was 97.9%. The smaller the prostate volume, the higher the possibility of a GS upgrade to ≥8 (OR: 0.94, 95% CI: 0.91-0.98, *P*=0.003). The higher the number of positive cores, the higher the possibility of a GS upgrade to ≥8 (OR: 3.16, 95% CI: 1.81-5.53, *P*<0.001). The higher the CRMP4 promoter methylation rate, the more likely a GS upgrade to ≥8 (OR: 1.43, 95% CI: 1.32-1.55, *P*<0.001; **Table 2**).

**Table 2:** Univariate and multivariate logistic regression to identify risk factors predicting a postoperative upgrade in GS to ≥8.

Variable	Univariable			Multivariable		
	OR	95%CI	p-value	OR	95%CI	p-value
**Age**	1.01	0.98-1.05	0.468	–	–	–
**PSA**	0.84	0.74-0.95	0.005	–	–	–
**prostate volume**	0.96	0.95-0.98	<0.001	0.94	0.91-0.98	0.003
**PSAD**	2.29	0.37-14.28	0.377	–	–	–
**No.of cores**	1.00	0.93-1.08	0.945	–	–	–
**Positive cores**	1.95	1.52-2.49	<0.001	3.16	1.81-5.53	<0.001
**CRMP4 value**	1.40	1.32-1.49	<0.001	1.43	1.32-1.55	<0.001
**Clinical T stage**						
T1	Reference	–	–	Reference	–	–
T2a	3.98	1.78-8.91	0.001	–	–	–

OR, odds ratio; CI, confidence interval; GS, gleason score; PSA, prostate specific antigen; PSAD, prostate specific antigen density; CRMP4, collapsin response mediator protein 4; Positive predictive value, 85.2%; Negative predictive value, 99.3%; Total predictive value, 97.9%.

#### 3.3.2 Comparison of the Predictive Performance of Different Pathologic Factors for a Postoperative GS Upgrade to ≥8 Based on ROC Analysis

**Figure 1:**
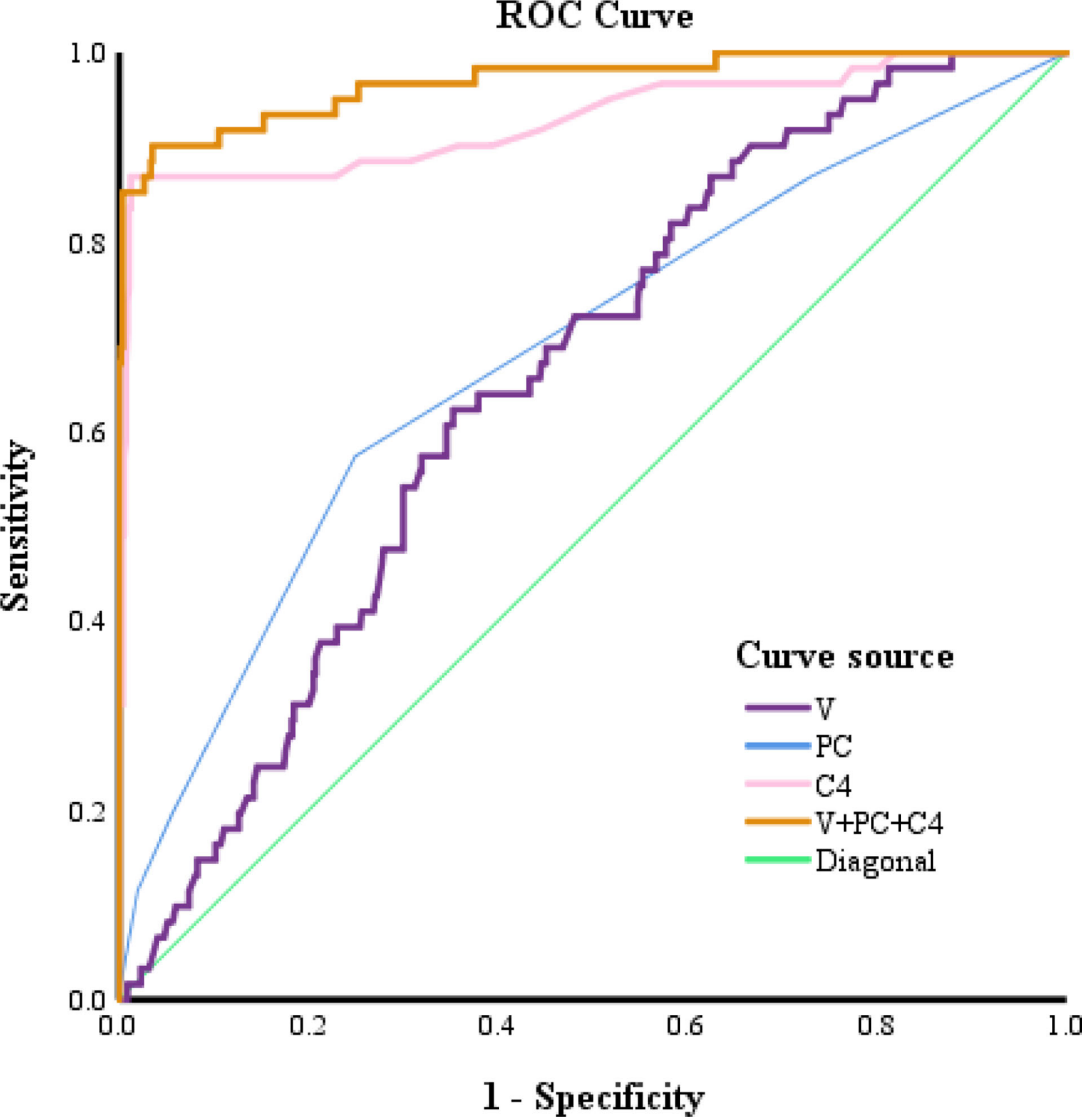
ROC curves for predicting a postoperative upgrade in GS to ≥8. V, prostate volume; PC, Positive cores; C4, collapsin response mediator protein 4.

A comparison of the ROC curves indicated that the CRMP4 promoter methylation rate had the highest accuracy in predicting a GS upgrade to ≥8. The cut-off value for the CRMP4 promoter methylation rate estimated from the ROC curve was 18.00%, above which a GS upgrade to ≥8 was more likely to occur. The sensitivity and specificity of the cut-off value were 86.9% and 98.8%, respectively. The area under the ROC curve was 0.929 (95% CI: 0.883, 0.976; *P*<0.001). The CRMP4 promoter methylation rate had a higher diagnostic accuracy. The cut-off value for the number of positive cores estimated from the ROC curve was 2, above which a GS upgrade to ≥8 was more likely to occur. The sensitivity and specificity of the cut-off value were 57.4% and 75.1%, respectively. The area under the ROC curve was 0.680 (95% CI: 0.605, 0.756; *P*<0.001). The number of positive cores had a lower diagnostic accuracy. The cut-off value for the prostate volume estimated from the ROC curve was 32.43 ml, below the value for which a GS upgrade to ≥8 was more likely to occur. The sensitivity and specificity of the cut-off value were 62.3% and 64.7%, respectively. The area under the ROC curve was 0.654 (95% CI: 0.591, 0.718; *P*<0.001). The prostate volume also had a lower diagnostic accuracy. The sensitivity and specificity for combining the above three factors in predicting a GS upgrade to ≥8 were 90.2% and 96.5%, respectively. The area under the ROC curve was 0.929 (95% CI: 0.943, 0.995; *P*<0.001). Combining the three factors had the highest diagnostic accuracy (**Figure 1**).

### 3.4 Comparison of the Postoperative Pathologic Evaluation Between Patients With and Without a Postoperative GS Upgrade to ≥8

**Figure 2:**
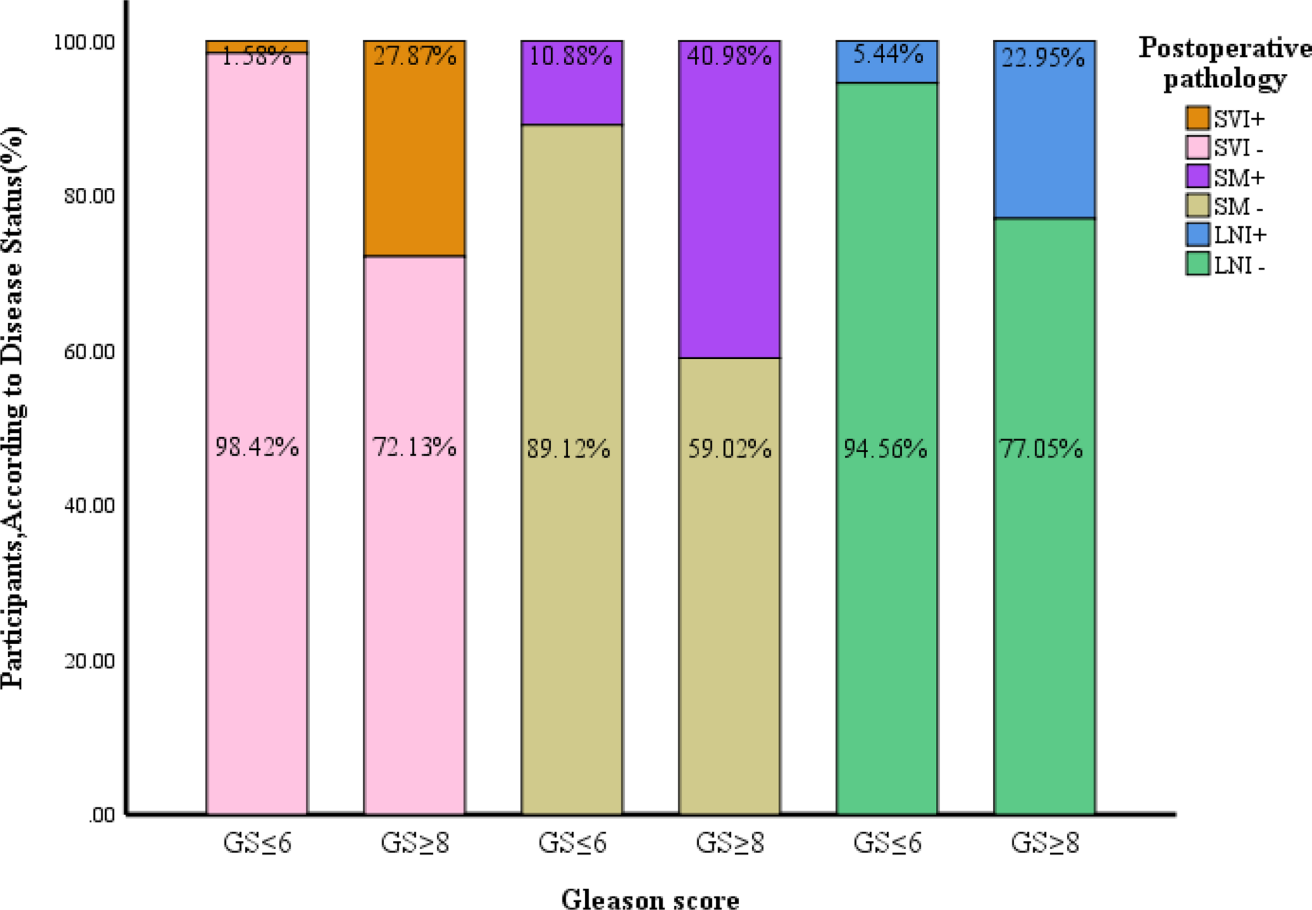
Comparison of pathological features between patients with a postoperative upgrade in GS to ≥8 and those without such an upgrade. GS, gleason score; SVI, seminal vesicle invasion; SM, surgical margin; LNI, lymph node invasion.

The pathologic T stage after surgery was more advanced. The seminal vesicle invasion rate, positive resection margin, pathologic T stage, and positive lymph node rate were higher in patients with a GS upgrade to ≥8 than in patients without an upgrade (*P*<0.001). The patients with seminal vesicle invasion accounted for 1.58% of all patients without a GS upgrade compared to 27.87% of patients with a GS upgrade to ≥8 (*P*<0.001). The patients with positive resection margins accounted for 10.88% of all patients without a GS upgrade compared to 40.98% in patients with a GS upgrade to ≥8 (*P*<0.001). The patients with positive lymph nodes accounted for 5.44% of all patients without a GS upgrade to 22.95% in patients with a GS upgrade to ≥8 (*P*<0.001; **Table 1** and **Figure 2**).

### 3.5 Comparison of the Prognosis of Patients With and Without a Postoperative GS Upgrade to ≥8

**Figure 3:**
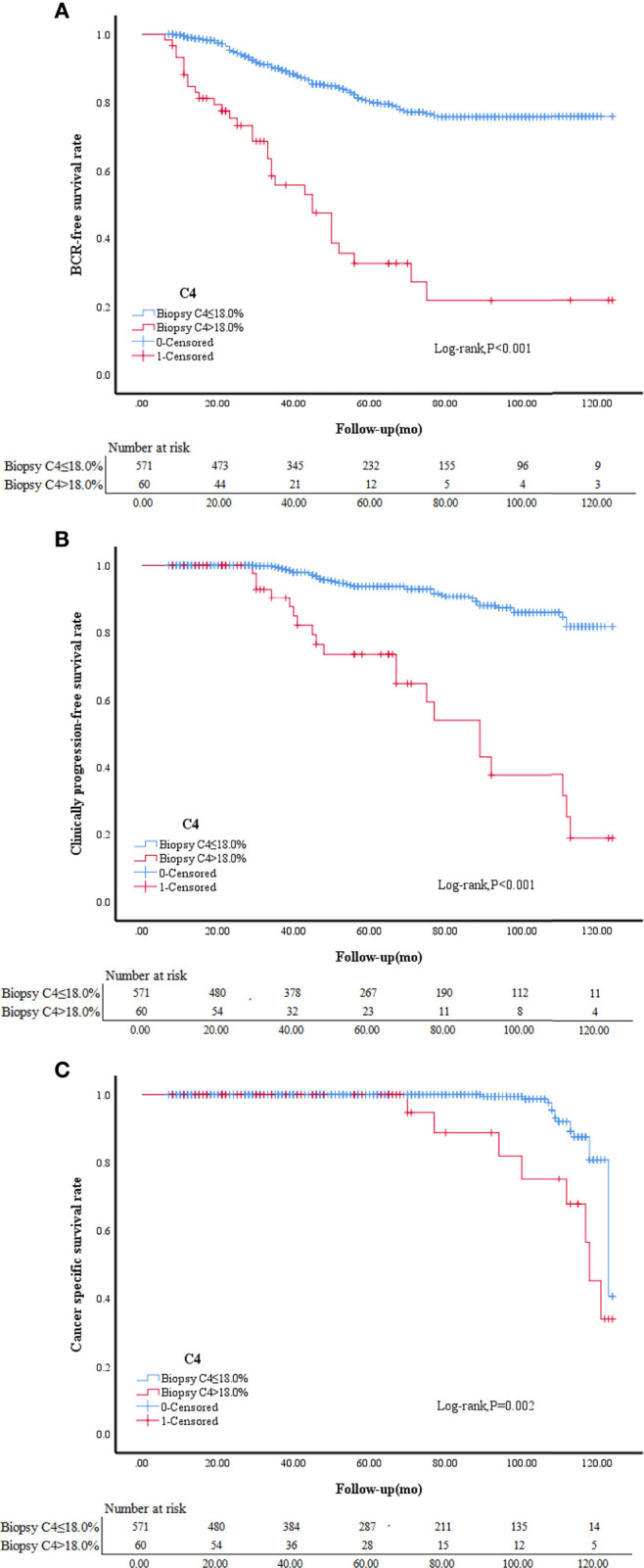
BCR-free survival in patients with biopsy C4 ≤ 18.0% and biopsy C4>18.0% **(A)**. Clinically progression-free survival in patients with biopsy C4 ≤ 18.0% and biopsy C4>18.0% **(B)**.Cancer specific survival in patients with biopsy C4 ≤ 18.0% and biopsy C4>18.0% **(C)**. C4, collapsin response mediator protein 4 methylation.

Kaplan-Meier survival analysis showed that the prognosis was worse in patients with a GS upgrade to ≥8 than patients without a GS upgrade. Patients with a BCR accounted for 15.44% of all patients without a GS upgrade compared to 57.38% of patients with a GS upgrade to ≥8 (*P*<0.001). The patients with clinical progression accounted for 6.67% of patients without a GS upgrade compared to 36.07% of patients with a GS upgrade to ≥8 (*P*<0.001). CS deaths accounted for 2.28% of all patients without a GS upgrade compared to 16.39% of patients with a GS upgrade to ≥8 (*P*<0.001; **Table 1**). Kaplan-Meier survival analysis showed that the BCR-free survival, progression-free survival, and CSS were worse in patients with CRMP4 methylation >18.0% than in patients with CRMP4 methylation ≤18.0% (*P*<0.001; *P*<0.001; *P*=0.002; **Figure 3**).

## 4 Discussion

Per NCCN guidelines (2), AS or RP without lymph node dissection is sufficient for low-risk PCa, while high-risk PCa patients may face a higher chance of lymph node metastases. Therefore, an RP with extended pelvic lymph node dissection (ePLND) is recommended to improve PCa prognosis. Our study demonstrated that the BCR-free survival, PFS, and CSS were worse in patients with a postoperative GS upgrade to ≥8 than in patients without an upgrade related to the pathologic features and biological behaviors of PCa; and selection of the surgical regimen. In an earlier study reported that the seminal vesicle invasion and positive lymph node rates are higher among patients with a GS update than in patients without an upgrade (19% vs. 5.4% and 9.6% vs. 2.3%; *P* = 0.001 and 0.008, respectively) (26). Another research showed the positive resection margin rate was higher in the patients with a GS upgrade than patients without an upgrade (33.0% vs. 11.2%; *P*<0.001) *(* 27). We also found that the seminal vesicle invasion, positive resection margin, and positive lymph node rates were higher among patients with a GS upgrade than in patients without an upgrade (27.87% vs. 1.58%, 40.98% vs. 10.88%, and 22.95% vs. 5.44%; *P <*0.001 for each). This indicated that the patients with a GS upgrade to ≥8 had adverse pathologic features. When selecting the surgical regimen, the surgeons should attach greater attention to those factors.

Studies have shown that an increase in the PSA and PSAD levels, number of positive cores, percentage of positive cores, and a decrease in the prostate volume and the number of positive cores predict a higher chance of GS upgrade (10, 11). Santok research.showed that a PSA of 10–20 ng/mL predicted a higher chance of a GS upgrade to ≥8 (15). In contrast, we found that age, PSA level, PSAD, and the number of biopsies did not correlate with a GS upgrade to ≥8 as we only included the low-risk PCa patients. Another possibility is that early screening for PCa is common in China, leading to generally low levels of PSA upon PCa patients. Our study indicated that a prostate volume<32.43 ml and a number of positive cores>2 were closely related to a GS upgrade to ≥8. Based on multivariate regression analysis, Qi reported that the smaller the prostate volume, the higher the possibility of a GS upgrade (P=0.033) (28). Prostate growth and differentiation are closely related to the dihydrotestosterone level. PCa patients with a smaller prostate volume have lower levels of testosterone and dihydrotestosterone and a limited secretion of prostatic growth factors, such as insulin and insulin-like growth factor (29). The low expression of these hormones results in a more adverse microenvironment, where only more invasive tumor cells can grow, and the occurrence of high-grade PCa may be promoted. Based on our results, the prostate volume predicted a GS upgrade and a GS upgrade to ≥8, which expresses great importance for an accurate evaluation of a GS upgrade in PCa. Other researchers have reported that the number of positive cores > 2 is an independent risk factor for a GS upgrade (*P*=0.045). This finding agrees with our result regarding the predictive performance of the number of positive cores for a GS upgrade to ≥8. The larger number of positive cores may reflect a broader distribution of cancer tissues and an excessive tumor burden. Because of the limitations in the biopsy technique, some cancer tissues with a local high GS may be missed, leading to an underestimation of GS based on biopsy. For this reason, the risk of a GS upgrade to ≥8 deserves extra attention in PCa patients with a larger number of positive cores. If conditions permit, the number of biopsies should be increased to avoid missing the cancer tissues with a local high GS. As noted in our study, the number of positive cores was equally important for accurately evaluating GS. This was the first study to identify the close connections between the prostate volume, the number of positive cores, and a GS upgrade to ≥8.

The CRMP family consists of CRMP1-5 (30–32), some studies have shown that CRMP4 expression is low in PCa. The methylation of CRMP4 promoter leads to a downregulation of CRMP4, which further promotes the invasion and metastasis of PCa and affects the prognosis (18, 24, 33). The next question is how CRMP4 promoter methylation is related to a GS upgrade. Our results showed that the higher the CRMP4 methylation rate, the more likely a GS upgrade to ≥8. Comparison of the ROC curves showed that the AUC for CRMP4 promoter methylation predicting a GS upgrade to ≥8 was 0.929. The sensitivity and the specificity were 86.9% and 98.8%, respectively, which were considerably higher than the prostate volume and number of positive cores. When the CRMP4 promoter methylation rate was >18.00%, the low-risk PCa patients were more likely to have a GS upgrade to ≥8 based on biopsy. According to the NCCN guidelines (2), an RP should be performed concomitantly with PLND for PCa patients with a GS≥8. We recommend RP and PLND for low-risk PCa patients with a preoperative CRMP4 promoter methylation rate > 18.00% based on preoperative biopsy. According to an earlier study, PCa patients with a CRMP4>15% are more likely to develop lymph node metastases, which agreed with our results (34). In addition, the combination of CRMP4 promoter methylation, prostate volume, and the number of positive cores had a much higher predictive accuracy than any other factor. The AUC was 0.969, and the sensitivity and specificity were 90.2% and 96.5%, respectively. Our model had the highest predictive accuracy for a GS upgrade to ≥8. Previously, few predictive models have been proposed for a GS upgrade to ≥8. The model proposed has an AUC of 0.924 by combining age, PSAD, PI-RADS score, and the number of positive cores to predict a GS upgrade (28). Incorporates the PSA level, the maximum percentage of cancerous components in each core, the PI-RADS score, and the number of positive cores,the AUC for predicting a GS upgrade is 0.90 (35). After eliminating the PI-RADS score, the AUC is only 0.64. These two models have higher predictive accuracy for a GS upgrade. Thus, the PI-RADS score is highly valuable in predicting a GS upgrade. A predictive model including the CRMP4 promoter methylation rate has even higher accuracy. Therefore, CRMP4 promoter methylation has an essential role in predicting a GS upgrade and greatly improves the model’s predictive power. Taken together, it is necessary to determine the CRMP4 promoter methylation rate based on a preoperative biopsy. The combination of the CRMP4 promoter methylation, prostate volume, and the number of positive core rates showed a much higher diagnostic accuracy for a GS upgrade and could better guide the clinical work.

We encountered the problem of multifocal tumors while collecting samples from prostate cancer patients. The distribution of prostate tumors presents multifocal incidence, and a variety of primary prostate cancers with different genomes and phenotypes may occur in the same patient, which brings difficulties in the diagnosis and treatment of prostate cancer (36, 37). In this study, the specimens of each enrolled patient were evaluated by professional pathologists for their classification, location and tumor load. In the biopsy tissue, the pathology report details the grade of tumor and the percentage of cancerous tissue at each needle. In the specimens after radical prostate cancer surgery, we selected the tumor tissues with the highest pathological GS grade for detection of CRMP4 methylation. Due to the multifocal nature of prostate cancer, we detected CRMP4 methylation in different parts of tumor tissues, and the results showed that there was no difference in the value of CRMP4 methylation in different parts of tumor in the same patient. By comparing the data of 61 patients upgraded to GS≥8, we found no significant difference in the methylation value of CRMP4 between the biopsy specimens and the postoperative specimens (mean 24.39% vs 24.79%, *P*=0.108). Our results showed that the methylation of CRMP4 was relatively stable in the same prostate cancer patient. Pathologists diagnose prostate cancer mainly by evaluating the epigenetics of the pathological biopsy section. However, this method has many affected factors, such as the limitations of biopsy, pathologist subjectivity, and the objectivity of prostate cancer pathology characteristics. Thus, many prostate cancer patients have a Gleason score upgrade after radical prostatectomy, which predicts a poor postoperative prognosis (38). In this study, we reported the methylation of CRMP4 predicts prostate cancer’s upgrading and predicts prostate cancer prognosis. However, pathological review in patients with low-grade cancer is mandatory to identify intraductal cancer, perineural invasion or other features that should be considered for adverse prognosis. (39) Overall, the methylation of CRMP4 is more reliable than GS in the diagnosis, treatment and prognosis of prostate cancer.

## 5 Limitations

This study mainly assessed the predictive value of CRMP4 methylation in predicting GS upgrade to ≥8, the limitations of this study are mainly reflected in two aspects. First, patients with low-risk prostate cancer diagnosed by biopsy are less likely to be upgraded to high-risk after surgery. We retrospectively collected multi-center data for 5 years. Among the 631 enrolled patients, only 61 (9.6%) patients had GS upgrade to ≥8 after surgery, the number of cases is relatively small, and we need to conduct in-depth research in a larger sample size. Secondly, the imaging data of all the enrolled patients were not obtained in this study, and the biopsy tissue of the prostate system only accounted for 0.01% of the total prostate volume. The obtained tumor tissue is probably not the most typical tumor foci, resulting in an underestimation of the biopsy GS score. With the widespread development of multiparametric MRI (40), we believe that MRI-guided targeted biopsy will certainly improve the accuracy of GS scores. CRMP4 methylation reflects a stable methylation frequency in tumor specimens. We believe that CRMP4 methylation detection in tumor specimens obtained by puncture can assist the existing technology to improve the accuracy of prostate cancer diagnosis.

## 6 Conclusions

Low-risk PCa patients with a CRMP4 promoter methylation rate > 18.00% based on preoperative biopsy were more likely to undergo a GS upgrade to ≥8 based on postoperative pathologic evaluation. The BCR-free survival, progression-free survival, and CSS were worse in patients with CRMP4 methylation >18.0% than in patients with CRMP4 methylation ≤18.0%.

## Data Availability Statement

The original contributions presented in the study are included in the article/**Supplementary Material**. Further inquiries can be directed to the corresponding authors.

## Ethics Statement

Ethical review and approval were not required for the study on human participants according to the local legislation and institutional requirements. Written informed consent from the participants’ legal guardian/next of kin was not required to participate in this study per the national legislation and the institutional requirements.

## Author Contributions

(I) Conception and design: X-PQ, Q-JL, and C-HY. (II) Administrative support: ZC and Y-MZ. (III) Provision of study materials or patients: X-PQ, Q-JL, C-HY, and ZC. (IV) Collection and assembly of data: X-PQ, Q-JL, JW, J-FC, KL, XC, JZ, Y-HP, Y-HL, S-CR, J-ML, W-PL, H-JQ, X-LY, C-YL, L-JQ, XG, and Y-SX. (V) Data analysis and interpretation: X-PQ, Q-JL, C-HY, ZC, and Y-MZ. (VI) Manuscript writing: X-PQ, Q-JL, C-HY, ZC, Y-MZ, JW, J-FC, KL, XC, JZ, Y-HP, Y-HL, S-CR, J-ML, W-PL, H-JQ, X-LY, C-YL, L-JQ, XG, and Y-SX. All authors contributed to the article and approved the submitted version.

## Funding

This work is supported by the following grants: National Natural Science Foundation of China (81902615); Postdoctoral Fund of the First Affiliated Hospital, Jinan University(809011); Postdoctoral Program of the International Training Program for Outstanding Scientific Research of Guangdong Province(2019); Leading Specialist Construction Project- Department of Urology, the First Affiliated Hospital, Jinan University(711006); Science and Technology Planning for Basic Research Project of Guangzhou-Municipal School (Institution) Joint Funding (Dengfeng Hospital) (20220102060116).

## Conflict of Interest

The authors declare that the research was conducted in the absence of any commercial or financial relationships that could be construed as a potential conflict of interest.

## Publisher’s Note

All claims expressed in this article are solely those of the authors and do not necessarily represent those of their affiliated organizations, or those of the publisher, the editors and the reviewers. Any product that may be evaluated in this article, or claim that may be made by its manufacturer, is not guaranteed or endorsed by the publisher.
