# Supplementary material for: CRMP4 CpG Hypermethylation Predicts Upgrading to Gleason Score ≥ 8 in Prostate Cancer
Source: Front Oncol. 2022 Mar 10;12:840950. doi: 10.3389/fonc.2022.840950 (PMC8960729; doi:10.3389/fonc.2022.840950)
Supplement: Supplementary file 1 [file DataSheet_1.doc]

**
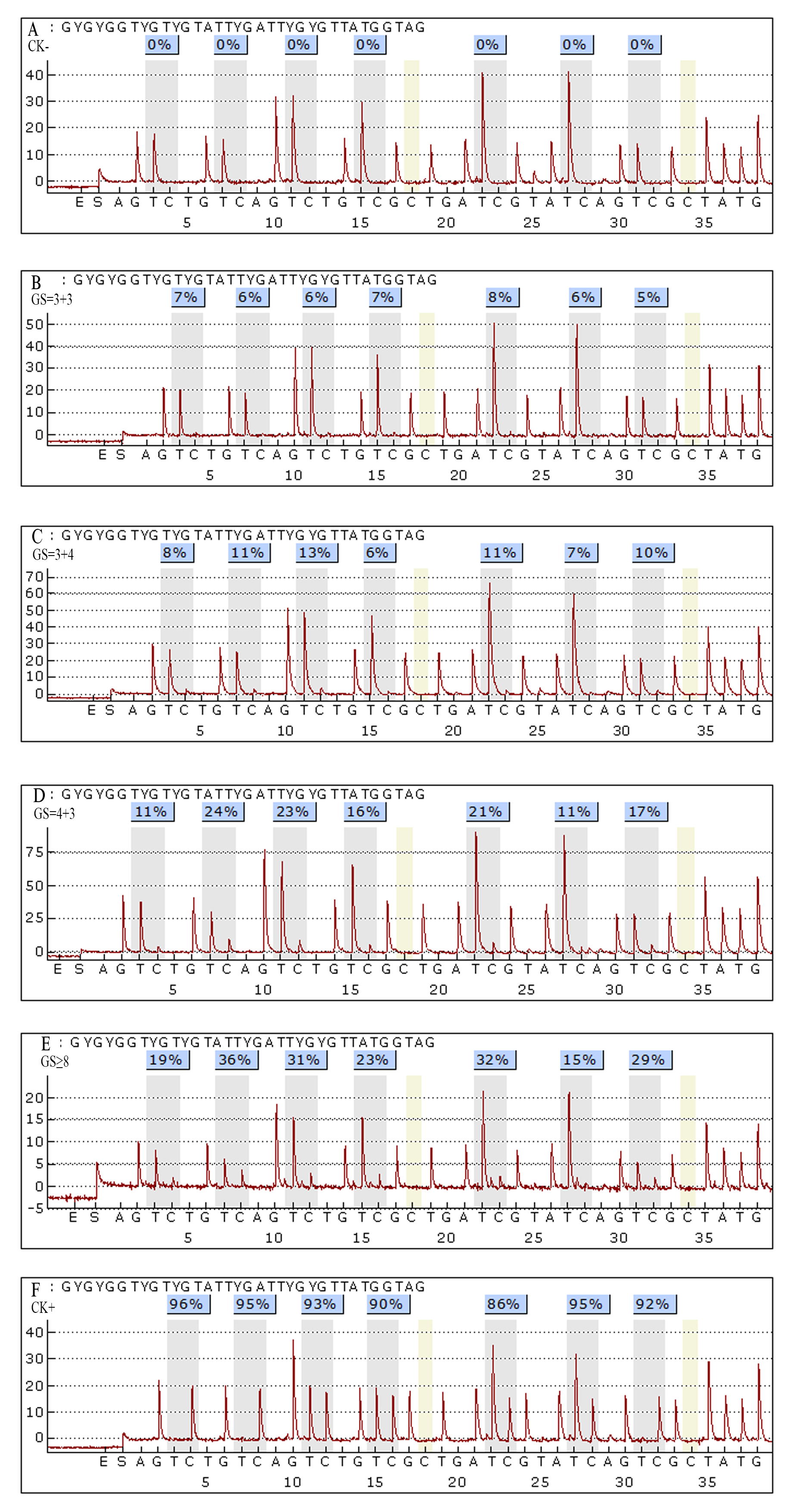
**

**Groups, negative and positive controls for pyrosequencing.** **A**. Negative(CK-) the genomic DNA were treated by 5-Aza, no CpG islands methylation was detected; **B.** GS≤3+3 CRMP4 methylation testing; **C.** GS=3+4 CRMP4 methylation testing; **D.** GS=4+3 CRMP4 methylation testing; **E.** GS≥8 CRMP4 methylation testing; **F.** Positive control(CK+), CpG islands were completely methylated.

**Information of CRMP4 promoter region base sequence：**

ATGGGGAGCAATAGCAGTTCTACTCCAAATCCATGGCTGCGCACCAGGAACGTGTCTGGGCTACTGCCTTCCCACTCCACAGGTGTTTTCTACTTTTTACTCACAGAGCAGCATTCATGTTCTTTCTTTGATTCTGAGACTCAGTCAGGCAGTGGAGTTTCTTTAACAAGATACTACTTGGATATTTTTTAAATACAGGGAATGGTTGAGTCCACTGCTACCGTGCCCCTTGCAGCCTCTGAGAGCGAGTCACGGCTTTCCATTTTCTAATGTGTATGTTCCGGGTCTGGGGACCCCAGCAGGCCCGCCA(66bp-G1-S1)GGTTGCAGAGTCGGACCAGTCTCCGGCGTCTGAA(51bp-G1-S2)GGGGCTGCGCCGCTGTTTACCACCCCGCGAGCAGCAGAGGCGGCGCCCAGCCCTCCTCTCGAA(60bp-G1-S3)CAAAGGCGCGGCCGCCGCATTCGACCCGCGCCATGGCAG(34bp-G1-S4)AGGAGAGCGGGTGCCGGGAGGGAAGCCGGGAACCGTCTCCATTCTGAAACTAGGGCGGGGAAG(56bp-G2-S5)GTAGGAGATCCGACGAGGAGCTGGCTGGGGGCGGCGGCGCCCGCAACTCGGCCCCGCGGCCGGGAGGGCGTA(63bp-G2-S6)GCTGATGCTCCCACCCCCGGGACTCAGCCTCCGCTCCCGAAAAGCCCCTCGCGCCCCCTGGTGGCTGTGGAGCAGCCTGACTCCGCAGCACTCGCGAATCAGAAAAAAAAAAAAAAGAAAGAAAAAGAAAAAGTGGGTGAGGCAGATGCAAAGAGGAAAATCAATAGGGATAAGAGAGAAGAGGGAGGAGTCGCAGATCAGCCATTCTTGTCTTATGGGGTCCCAGCTGGCGCAGCAAAAGAAAAAAAAAAACAAAACCCGGGCGTGAACGCGGGTGGAGGAGGGTGTGCGTGGGGGGGCGGGGGTGAAAGGGGGACCGGGGAGATTTACTATTGTCTCTGGCAGCCGCCGCGGGAGCCCGGGAGGGGGGCGGAGGCGGGCGGAGGCGGCGGCGCGGCCAGCGCACCATTCACTCCACCTGATCTCGGGGCGCTGTGCGCTGAGGAAGGCGCGGGCGAGCCGGAGCAGAAGAAGGAGGGAGGGAGCCAGCCGCTGCAGCCACCACCGCCACCATG

**CRMP4 promoter region(upstream of the transcription start site:Y)**

**(CG YG, C t)：**

ATGGGGAGtAATAGtAGTTtTAtTttAAATttATGGtTGYGtAttAGGAAYGTGTtTGGGtTAtTGttTTtttAtTttAtAGGTGTTTTtTAtTTTTTAtTtAtAGAGtAGtATTtATGTTtTTTtTTTGATTtTGAGAtTtAGTtAGGtAGTGGAGTTTtTTTAAtAAGATAtTAtTTGGATATTTTTTAAATAtAGGGAATGGTTGAGTttAtTGtTAtYGTGttttTTGtAGttTtTGAGAGYGAGTtAYGGtTTTttATTTTtTAATGTGTATGTTtYGGGTtTGGGGAttttAGtAGGttYGttA(66bp-G1-S1)GGTTGtAGAGTYGGAttAGTtTtYGGYGTtTGAA(51bp-G1-S2)GGGGtTGYGtYGtTGTTTAttAtttYGYGAGtAGtAGAGGYGGYGtttAGtttTttTtTYGAA(60bp-G1-S3)tAAAGGYGYGGtYGtYGtATTYGAttYGYGttATGGtAG(34bp-G1-S4)AGGAGAGYGGGTGtYGGGAGGGAAGtYGGGAAtYGTtTttATTtTGAAAtTAGGGYGGGGAAG(56bp-G2-S5)GTAGGAGATtYGAYGAGGAGtTY(rs116337841)GtTGGGGGYGGYGGYGttYGtAAtTYGGtttYGYGGtYGGGAGGGYGTA(63bp-G2-S6)GtTGATGtTtttAttttYGGGAtTtAGttTtYGtTttYGAAAAGttttTYGYGtttttTGGTGGtTGTGGAGtAGttTGAtTtYGtAGtAtTYGYGAATtAGAAAAAAAAAAAAAAGAAAGAAAAAGAAAAAGTGGGTGAGGtAGATGtAAAGAGGAAAATtAATAGGGATAAGAGAGAAGAGGGAGGAGTYGtAGATtAGttATTtTTGTtTTATGGGGTtttAGtTGGYGtAGtAAAAGAAAAAAAAAAAtAAAAttYGGGYGTGAAYGYGGGTGGAGGAGGGTGTGYGTGGGGGGGYGGGGGTGAAAGGGGGAtYGGGGAGATTTAtTATTGTtTtTGGtAGtYGtYGYGGGAGttYGGGAGGGGGGYGGAGGYGGGYGGAGGYGGYGGYGYGGttAGYGtAttATTtAtTttAttTGATtTYGGGGYGtTGTGYGtTGAGGAAGGYGYGGGYGAGtYGGAGtAGAAGAAGGAGGGAGGGAGttAGtYGtTGtAGttAttAtYGttAttATG

**CRMP4 primers:**

| **CRMP4 Region** A | CZ-G1-F1 | ATAGGGAATGGTTGAGTTTATTGT |
| --- | --- | --- |
|  | CZ-G1-R1-Bio | Biotin-ACCCCCTCTCCTCTACCATA |
|  | CZ-G1-S1 | GTTTTTTGTAGTTTTTGAGA |
|  | CZ-G1-S2 | GTTTAGGGTTTGGGGA |
|  | CZ-G1-S3 | TTTAGGAGTTTGAAGGG |
|  | CZ-G1-S4 | AGTTTTTTTTTAGAATAAAG |
| **CRMP4 Region** B | CZ-G2-F2 | AGGGTTTGGGGATTTTAGTAGGT |
|  | CZ-G2-R2-Bio | Biotin-TCCCCAAAATAAAAACATCAACT |
|  | CZ-G2-S5 | TAGAGTTATGGTAGAGGAGAG |
|  | CZ-G2-S6 | GGGGAAGGTAGGAGAT |
