# Supplementary figures and images for: CRMP4 CpG Hypermethylation Predicts Upgrading to Gleason Score ≥ 8 in Prostate Cancer
Source: Front Oncol. 2022 Mar 10;12:840950. doi: 10.3389/fonc.2022.840950 (PMC8960729; doi:10.3389/fonc.2022.840950)

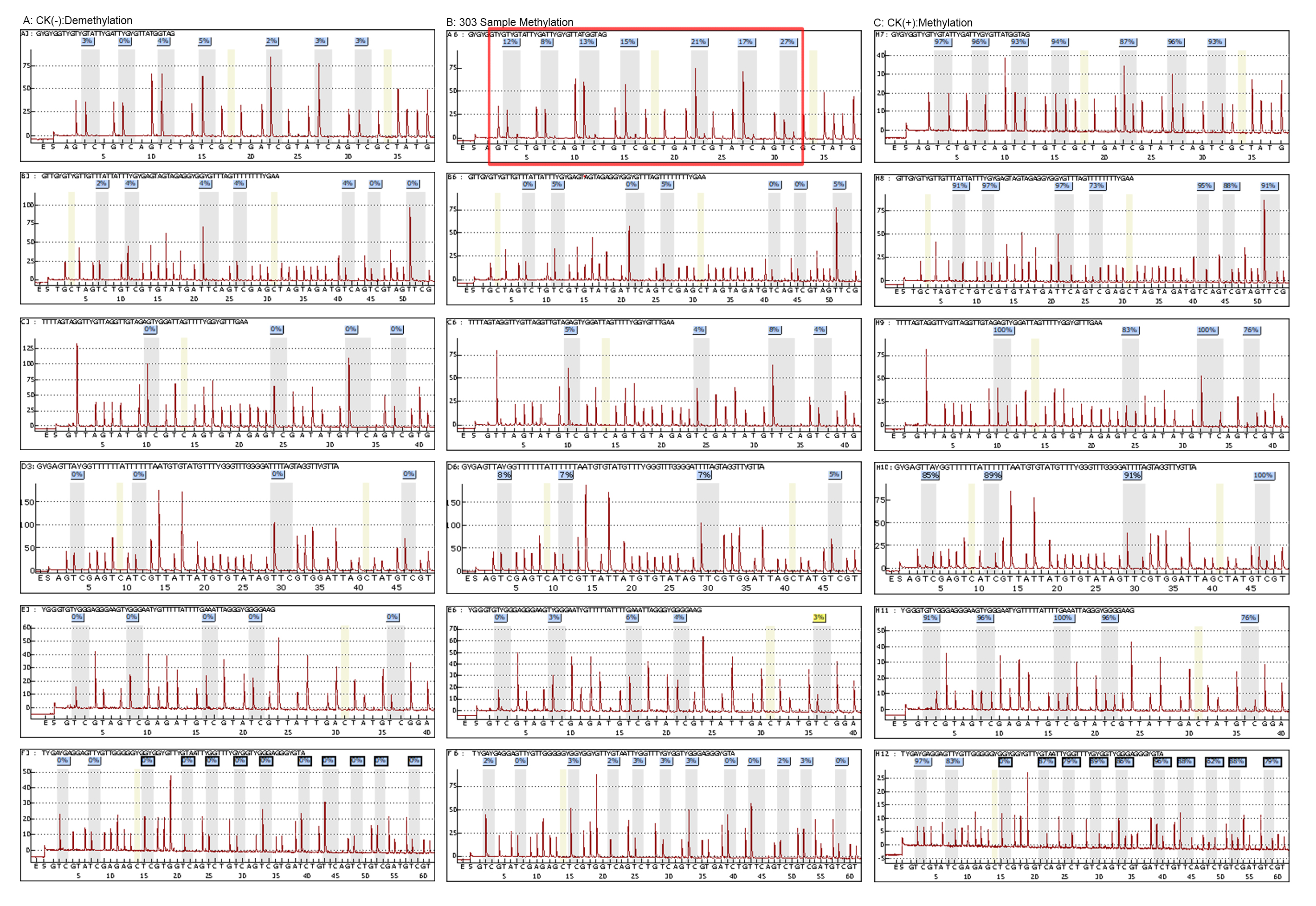

Supplement: Supplementary file 2 [file DataSheet_2.docx]
